# Supplementary material for: Are carbon emissions trading and green financial instruments synergistic? -Comprehensive quantitative research based on content analysis
Source: PLoS One. 2024 Mar 7;19(3):e0298601. doi: 10.1371/journal.pone.0298601 (PMC10919601; doi:10.1371/journal.pone.0298601)
Supplement: S1 Data — (ZIP) [file pone.0298601.s001.zip › Data/地级市Z_F工作报告.zip!/║╙─╧/╓▄┐┌╩╨/└┤╫╘╣1⁄2╓┌║┼í╛┬φ┐╦╩2╛▌═°í┐_2014.docx]

各位代表：

　　现在，我代表市人民政府，向大会报告工作，请予审议，并请各位政协委员和其他列席人士提出意见。

　　一、2013年工作回顾

　　过去一年，面对复杂严峻的经济形势和艰巨繁重的发展任务，在省委、省政府和市委的正确领导下，全市上下深入贯彻落实党的十八大、十八届二中、三中全会和市委三届六次全会精神，克难攻坚，积极作为，各项工作取得了新的成绩。总体讲，全市经济运行稳中有进，各项事业全面发展，民生福祉持续改善，社会大局和谐稳定。初步核算，全市生产总值完成1790.7亿元，规模以上工业增加值完成672.8亿元，固定资产投资（不含农户）完成1151.6亿元，社会消费品零售总额完成756.8亿元，公共财政收入达到76亿元，城镇居民人均可支配收入达到18046元，农民人均纯收入达到6950元，节能减排完成省政府下达的目标任务。具体表现可以概括为“十个较快增长、六个明显提升”。

　　十个较快增长：

　　初步核算，生产总值增长9.3%，高于全省0.3个百分点；公共财政收入增长26.5%，高于全省8.2个百分点；规模以上工业增加值增长16.4%，高于全省4.4个百分点；固定资产投资（不含农户）增长23.7%，高于全省0.5个百分点；规模以上工业实现利润增长30.3%，高于全省17.3个百分点；工业用电量增长13.4%，高于全省10.5个百分点；产业集聚区固定资产投资增长26.8%，高于全省1.8个百分点；实际利用外商直接投资增长21.7%，高于全省10.7个百分点；外贸进出口总额增长38.2%，高于全省22.3个百分点；财政民生支出增长12.6%，高于全省0.7个百分点。全市主要经济指标增速连续两年高于全省平均水平，经济总量由全省第7位跃居第5位。

　　六个明显提升：

　　一是粮食生产再创历史新高，农业生产能力明显提升。粮食生产克服多种自然灾害再获丰产丰收，总产量达到158.8亿斤，稳居全省第一位，我市再次被评为全国粮食生产先进市，太康等8个县市被评为全国粮食生产先进单位。新建高标准粮田156.7万亩，粮食生产能力稳步提升。培育省级农业产业化集群13个，新注册农民合作社4056家、家庭农场654个，新增土地流转面积43万亩，农业生产经营组织化、规模化、专业化步伐加快。新建和改扩建大中型标准化养殖场（区）195家，规模养殖比重达到85%以上，畜牧业总产值达到225亿元；完成林业生态建设面积9.6万亩，全市林木覆盖率达到25％，林业总产值突破100亿元，农业结构进一步优化。完成153个贫困村整村推进任务，18.5万农村贫困人口实现稳定脱贫。新解决84.6万农村居民和农村中小学师生饮水安全问题，新修农村公路345公里，农村生产生活条件持续改善。

　　二是工业增速位居全省前列，产业集聚效应明显提升。全市规模以上工业企业达到1124家，主营业务收入、增加值、实现利润增速分别居全省第一、第二、第三位。食品加工、纺织服装、医药化工三大支柱产业增加值达到406.3亿元，占全市规模以上工业增加值总量的60.4%，依然是拉动工业经济增长的主导力量。全市11个产业集聚区规模以上工业企业达到429家，增加值增长25.7%，主导产业优势更加突出，集群发展趋势更加明显。全市省级以上企业技术中心达到28个，国家级高新技术企业达到16家，实施企业技术改造项目79个。新认定河南省著名商标23件，新增河南名牌产品27个，企业品牌和市场竞争力稳步提升。积极引导企业实施战略重组，百年康鑫、金丹乳酸等5家企业股权融资9.3亿元，金丝猴集团与北美最大的巧克力制造商美国好时公司、大用集团与麦当劳全球最大的供应商美国福喜集团实现战略合作，为我市工业发展注入了新的活力和动力。

　　三是中心城区建设迈出新步伐，城乡发展质量明显提升。周口市城市总体规划修编纲要、水系景观规划编制、沙颍河景观规划编制、商务中心区和特色商业区规划编制等基本完成，中心城区“十大建设”全面启动。周口城乡一体化示范区（周口新区）建设步伐加快，港口物流产业集聚区“三纵一横”路网建设扎实推进，东外环路及跨沙颍河大桥完成年度建设计划，市行政中心、招商大厦、沙颍河城区段治理一期工程等项目进展顺利。新火车站和站前广场建成投用，中州路等道路改造基本完成。实施15个城中村改造项目，完成房屋征迁面积144万平方米。6个标准化小型菜市场及小型餐饮市场开工建设。第一期城市投资债券顺利发行。探索和理顺城市管理体制，推行网格化管理，市容市貌逐步改善。周淮快速通道、周西快速通道（西华段）实现通行，组团城市融合发展趋势逐步增强。县城、小城镇和新农村建设有序推进，发展质量和活力稳步提升。城镇化率达到35.24%，较上年提高1.8个百分点。

　　四是招商引资实现新突破，对外开放水平明显提升。深入实施“招商引资项目落实年”和“承接产业转移突破年”活动，改变招商方式，提高招商实效，全年签约亿元以上项目144个，实际到位省外资金379亿元，增长20%。投资60亿元的中部鞋业轻工文化产业园、投资60亿元的正大集团全产业链养殖加工、投资75亿元的申鹭达建材产业园等一批龙头型、基地型项目相继落户我市。鼓励企业走出去创业发展，新增金丹欧洲有限责任公司等3家境外投资企业，对外合作取得了新进展。持续推进“河南省制鞋产业出口基地”和“河南省食品调味品出口基地”建设，引进一批出口型项目，做大一批出口型企业，积极扩大对外贸易，全年实现出口总值5.9亿美元，增长57%。

　　五是项目建设势头强劲，投资拉动能力明显提升。全市亿元以上在建项目553个，完成投资809.2亿元，增长42.2%，高于全省增速19.6个百分点。189个市级重点项目完成投资557.4亿元，是年度计划的116.2%。备受关注的郑州经周口至合肥高铁项目已正式纳入国家“十二五”铁路建设修编规划，省政府已决定全面启动前期工作，通用机场项目已获国家有关部门批复。福喜集团熟食加工、娃哈哈饮料生产项目投产运营，新周（新郑机场至周口）高速公路全线开工建设，鸿闽纺织集群服装加工、南水北调（周口段）配套工程、家乐福商业综合体等一批项目快速推进，和合纺织高档面料印染加工、沙颍河周口至漯河段航运开发、华耀商贸物流城、碧桂园城市综合体等一批项目进展顺利。投资增长保持强劲势头，为全市经济发展提供了有力支撑。

　　六是民生福祉持续改善，社会管理水平明显提升。持续加大民生投入力度，全市财政民生支出280亿元，占财政支出的78.1%，较上年提高1.5个百分点，十项重点民生工程全面完成。实施积极的就业政策，完成职业技能培训30.5万人次，新增城镇就业10.6万人，新增农村劳动力转移就业10.7万人。社会保障能力不断提高，发放养老金42亿元、低保金9.5亿元，发放率达100%，农村五保老人供养标准提高720元。开工建设保障性住房20921套，建成14259套，新改造农村危房1.45万户。财政预算安排教育支出94.4亿元，增长17.6%，占公共财政支出的26.3%；新建和改扩建学校887所，教学条件逐步改善；实施中心城区教育振兴工程，支持周口一高打造中原名校，建成投用文昌中学，启动“一校两园” 建设；高招本科上线率高出全省平均水平，其中有51名新生被清华、北大录取，持续在全省领先；6个县市被评为职教强县，职业技术教育取得新进展。推进基本公共卫生服务均等化，新农合参合率稳定在99%以上，基层医疗卫生服务体系逐步健全，国家基本药物制度实现全覆盖。全民健身运动广泛开展，体育设施进一步完善。稳定低生育水平，人口自然增长率控制在4.76％。。公共文化服务体系建设扎实推进，文化惠民工程顺利实施，“舞台艺术送农民”演出170余场，送戏下乡演出2500余场，农村电影放映5万余场，豫剧《山城母亲》等5个精品项目荣获国家级大奖。旅游业稳步发展，实现总收入80.9亿元，增长16.5%。科技创新步伐加快，荣获省科技进步二等奖1项、三等奖3项，申请专利878件。加强环境保护和生态建设，实施主要污染物总量控制预算管理，PM2.5监测系统建成投用。实现管道输气“县县通”，清洁能源普及率稳步提高。强化监管措施，安全生产和食品药品安全形势稳中向好。创新社会治理和群众工作机制，信访形势总体平稳，平安建设富有成效。老龄、妇女儿童、残疾人、慈善等各项事业全面发展，社会管理水平不断提高，社会大局保持和谐稳定。

　　一年来，市人民政府自觉接受人大及其常委会的法律监督、工作监督和政协的民主监督以及社会监督，办理人大代表建议116件、政协提案350件。加强政府自身建设，全面推进依法行政，行政效能和公务员素质不断提高。强化监督制约，廉政建设取得新成效。开展第四届周口市道德模范评选等创建活动，精神文明建设扎实推进。积极支持驻周解放军和武警部队建设，落实军人抚恤优待政策，做好军队转业干部和退役士兵就业安置、民兵预备役、人民防空工作，军政军民团结进一步增强，连续四届荣获省级双拥模范城称号。统计、审计、供销、物价、工商、海关、民族、宗教、史志、对台、外事、侨务、哲学社会科学、防震减灾和对口援疆等各方面工作都取得了新的成绩。

　　各位代表！

　　上述成绩的取得，是省委、省政府和市委科学决策、正确领导的结果，是市人大及其常委会依法监督、大力支持的结果，是市政协建言献策、积极帮助的结果，是各级各部门抢抓机遇、勇于作为的结果，是全市人民团结一心、艰苦奋斗的结果。在此，我代表市人民政府，向工作在各条战线上的工人、农民、知识分子、干部、人民解放军指战员、武警消防官兵、公安民警致以崇高的敬意！向所有关心、支持周口发展的中央和省驻周单位、社会各界人士及海内外朋友表示诚挚的感谢！

　　各位代表！

　　在充分肯定成绩的同时，我们也清醒地认识到，全市经济社会发展还面临着不少困难和问题。一是个别经济指标没有完成年度目标。生产总值增长9.3%，社会消费品零售总额增长13.9%，虽高于全省平均增速，但受主客观等多种因素影响，仍未能达到市三届人大三次会议确定的10%和16%的预期目标。二是项目建设管理存在不足。一些重点项目，包括“双十五工程”中的部分项目，由于前期论证工作不扎实和后续跟进工作不到位等原因，进展不理想，有的没有完成时序进度，个别项目未能开工建设。三是影响我市经济发展的深层次矛盾仍然比较突出。经济总量小，人均水平低，经济发展的基础还比较薄弱。产业结构不合理，农业占比大，比较效益低；高成长性产业、战略性新兴产业刚刚起步，工业质量和效益不高；服务业发展滞后，带动就业、贡献税收较少。大项目、大企业少，对经济社会发展的支撑作用不强。贷款增量小，存贷比低，金融支持地方经济发展的能力较弱。城镇化水平低，中心城区引领带动能力不强。四是社会治理水平有待提升。公共服务水平较低，征地拆迁、环境保护、医疗卫生、食品药品安全等领域还存在一些群众反映比较强烈的问题，特别是中心城区的建设管理水平不高，与群众的期待差距较大。五是政府职能转变不到位。个别部门缺乏担当意识，服务不主动，作风不扎实，办事环节多，工作效率低，“庸懒散浮软”和“躁假浅怨混”等现象不同程度存在，发展环境有待进一步优化。对此，我们一定保持清醒忧患，积极加以应对，认真加以解决。

　　二、2014年工作总体要求和目标任务

今年是贯彻落实党的十八届三中全会精神和全面深化改革的重要一年，也是完成“十二五”规划的关键一年。持续改善的宏观经济环境和中央、省一系列重大决策，将为周口的发展带来更多机遇。中央更加注重农业和粮食，更加注重区域协调发展，更加注重加快推进中西部地区城镇化，周口作为传统农区、中西部贫困地区和大别山扶贫连片开发重点区，将会得到更多的政策支持和资金投入。

省委、省政府实施“三大国家战略规划”，推进“一个载体、三个体系”建设，打造“米”字形城镇密集带和快速铁路网，这些战略举措必将为周口发展带来一系列重大机遇。这里特别需要指出的是，市委确立了周口崛起方略，加快发展的思路更加清晰，目标更加明确，措施更加完善。周口已经站在了新的发展起点上，只要我们始终保持奋发有为的精神、务实重干的作风，用足用好各项政策，抓紧抓牢各种机遇，周口崛起大有希望，周口前景十分看好！

　　今年政府工作的总体要求是：全面贯彻落实党的十八大和十八届二中、三中全会精神，以及省委、省政府和市委决策部署，紧紧围绕周口崛起、富民强市、全面建成小康社会的总目标，把改革创新、扩大开放贯穿于经济社会发展各个领域各个环节，全面实施周口崛起方略，持续推进粮食生产核心示范区、产业集聚区、中心城区三区统筹发展，力争在项目建设、开放招商、中心城区发展、民生改善、改革创新上取得突破性进展，调中求进，改中创新，转中向好，稳中有为，切实提高经济发展的质量和效益，促进经济持续健康快速发展、社会和谐稳定。

　　今年经济社会发展主要预期目标是：在提高质量和效益的基础上，生产总值增长9.5%；地方公共财政预算收入增长13%；全社会固定资产投资增长22%；社会消费品零售总额增长14%；外贸进出口总额增长10%；实际利用外商直接投资增长10%；城镇居民人均可支配收入、农民人均纯收入实际增长9%；城镇新增就业7万人以上；人口自然增长率控制在6.5％。以内；居民消费价格控制在3.5%左右；节能减排完成省定目标任务。

　　贯彻总体要求，实现发展目标，必须按照中央、省委、省政府和市委的决策部署，立足周口实际，实施“总方略”，把握“主基调”，统筹“三重点”，实现“五突破”。实施“总方略”，就是以周口崛起、富民强市、全面建成小康社会为总目标，健全三大体系，坚持五大原则，构建七大载体，抓好九大攻坚，开展十大建设，努力在中原崛起、河南振兴的伟大进程中有更大作为。把握“主基调”，就是调中求进、改中创新、转中向好、稳中有为。调中求进，就是调整经济结构，把推动发展的立足点切实转到提高质量和效益上来，打造周口经济升级版。改中创新，就是全面深化改革，通过改革解放思想、激活体制机制，使各项工作呈现出新面貌。转中向好，就是通过转变思想观念、转变发展方式、转变领导方式，推动经济社会发展继续保持好的态势、好的趋势、好的气势。稳中有为，就是正确处理改革发展稳定的关系，保持政治稳定、经济稳定、社会稳定、舆情稳定，让百姓安居乐业，在稳定中实现跨越发展。统筹“三重点”，就是统筹推进粮食生产核心示范区、产业集聚区、中心城区建设，保障国家粮食安全，壮大载体支撑，统筹城乡协调发展。实现“五突破”：一是实现项目建设新突破。把项目建设摆在经济工作的核心位置，充分发挥投资拉动经济增长的关键作用，持续做大经济总量。二是实现开放招商新突破。充分发挥招商引资“一举应多变、一招求多效”的作用，继续大力实施开放招商，承接产业转移，夯实产业基础，做优产业结构。三是实现中心城区发展新突破。充分发挥新型城镇化“一发动全身”的带动作用，加快中心城区建设，进一步确立豫东南区域中心城市地位，引领我市新型城镇化快速推进。四是实现民生改善新突破。加大民生投入，办好民生实事，让人民群众更多更公平地享受改革发展成果。五是实现改革创新新突破。全面落实各项改革部署，转变职能、提高效率，优化环境、促进公平，激发经济社会发展活力。

　　各位代表！

　　上述目标任务的确立，再次吹响了富民强市新征程的冲锋号角。满载着群众对美好生活的期盼，担负着周口崛起的重任，我们一定恪尽职守、拼搏进取，在新的一年里，凝聚全市人民的智慧和力量，奋力开创周口追赶跨越发展的新局面！

　　三、2014年重点工作

　　今年，在科学统筹、全面发展的前提下，重点做好十个方面的工作。具体讲，就是“十个着力推进”。

　　（一）着力推进项目建设扩总量

　　坚持把项目建设作为做大总量、加快发展的主抓手，继续实施项目带动战略不动摇，加大项目建设力度，保持投资拉动经济增长的强劲态势。

　　突出抓好以“双十五工程”和中心城区项目为核心的182个市重点项目，切实提高项目资金到位率、开工建设率和建成投用率。加快推进碧桂园城市综合体、华耀商贸物流城等投资超百亿元的大项目，提升大项目对经济社会发展的带动能力；加快推进圣光医药医疗器械、金丝猴高档巧克力、润泰服装等工业项目，力争早日投产达效；加快推进新周高速、郸淮一级公路、沙颍河周口段航运开发项目，尽快启动通用机场、城乡电网改造升级、周项和郸沈快速通道等基础设施项目；完成一批农业和水利基础设施项目、扶贫开发项目，促进项目建设在经济领域全覆盖，保持经济社会发展的持久动力。

　　加强项目的谋划和推介，注重增加新开工项目比重、工业项目比重、中心城区项目比重、重点项目在全社会固定资产投资中的比重。积极研究国家投资政策、产业政策、区域开发政策，主动对接，争取有更多项目列入国家支持行列和省重点项目。配合国家和省做好郑合高铁、沙颍河周口下游至省界航道升级等项目前期工作，努力实现重大项目建设新突破。围绕中心城区“十大建设”，谋划推进“四个一批”项目，即一批城市基础设施项目、一批工业项目、一批商贸物流项目、一批社会民生项目，加快中心城区发展步伐。

　　加强项目建设管理，建立重点项目台账，将项目年度建设任务明确化、具体化，将所享受的优惠政策与投资额度、建设进度挂钩，建立规范有序、有进有退的项目建设管理长效机制。认真落实项目联席会议、联审联批、全程代办及重点项目周报告、旬通报、月点评等制度，继续采取领导分包、现场办公、观摩点评、督查督导等办法，全面提高项目建设管理水平。

　　（二）着力推进农业发展稳基础

　　自觉履行保障国家粮食安全的政治责任，持续推进“两区一体系”建设，创新农业经营体制机制，加快提升农业发展水平。

　　扎实推进粮食生产核心示范区建设，继续整合涉农资金，统筹水、电、林、路等基础设施建设，进一步增强粮食综合生产能力，力争今年粮食生产再上一个新台阶。加快实施高标准粮田“百千万”工程，新建高标准粮田150万亩以上。实施高标准粮田建设提升工程，重点支持商水30万亩整片开发示范区建设。

　　深入推进现代农业示范区建设，以现代畜牧业和特色高效农业为主攻方向，调整农业结构，提升产业化发展水平。围绕延长链条、完善标准、龙头带动、品牌提升，继续实施农业产业化集群培育工程。大力发展畜牧养殖业，年内新建、改扩建大中型标准化养殖场（区）180个以上。重点支持西华培育以大用集团、牧原集团为龙头的畜牧产业集群，打造全省畜牧养殖加工强县。抓好50个标准化农产品生产示范基地建设，年内新增无公害农产品、绿色食品、有机食品和农产品地理标志产品30个以上。重点支持扶沟20个标准化蔬菜基地建设，提升生产能力和品牌形象。

　　积极发展都市生态农业，创新现代农业新型业态。加快推进周口现代农业（示范）研究开发基地建设，打造集种苗培育、观光旅游于一体的都市生态农业基地。围绕城乡居民日常生活需求，在中心城区和有条件的县城周边，规划建设一批都市生态农业休闲园区，大力发展蔬菜、果品等绿色高效休闲农业，着力培育农业发展的新亮点。

　　创新农业经营体制机制，积极培育新型农业经营主体。规范发展农民合作社，年内示范社比重达到15%以上。大力发展家庭农场和种植大户，支持社会资本到农村从事农业开发，鼓励承包经营权向专业大户、家庭农场、农民合作社、农业企业流转，年内新增土地流转面积50万亩以上，进一步提高农业发展的规模化、集约化和专业化水平。

　　加快农业服务体系建设，全面落实强农惠农富农政策，不断改善农村生产生活条件。大力推广农业机械新技术，依托市农科院技术优势，继续推进良种良法推广应用，充分发挥天气雷达在灾害预警、人工增雨方面的作用，进一步提高农业科技保障水平。规范农资市场秩序，切实保护农民利益。实施“乡村畅通”工程，新改建农村公路460公里。再解决70万农村居民和农村中小学师生饮水安全问题。扎实推进扶贫开发，用好大别山连片扶贫开发的政策机遇，争取项目支持，注重产业扶贫，切实增强贫困地区发展的内生动力。年内完成140个贫困村整村推进任务，实现20万农村贫困人口稳定脱贫。

　　（三）着力推进工业发展壮支撑

　　坚持“工业强市”战略不动摇，持续做大总量，做优结构，提升效益，促进集群发展，进一步增强对经济发展的主导和支撑作用。

　　加强载体建设，完善功能配套，提高产业集聚区发展水平，强化集约节约发展。探索建立产业集聚区企业准入和退出机制，提高企业入驻的技术、环保、投资强度、税收等标准，淘汰落后产能，清退“双低”（低就业、低效益）企业和闲置土地企业，防止个别企业以投资建厂为名，行圈占土地之实，切实提高单位土地产出效益。今年起，投资亿元以下的企业，不再单独供地，一律进入标准化厂房。

　　改造提升传统支柱产业，以调整、优化和提升为方向，做大做强食品加工、纺织服装、医药化工三大支柱产业，促进产业链条向高端延伸。加快培育一批销售收入超百亿、超千亿的龙头企业和产业集群，推动工业经济向现代产业集群转型升级。以市经济开发区和沈丘、西华等产业集聚区为依托，持续打造千亿元食品加工产业集群；以扶沟、太康、商水等产业集聚区为依托，持续打造千亿元纺织服装产业集群；以项城、郸城等产业集聚区为依托，持续打造百亿元医药化工产业集群；以市经济开发区、高新技术产业开发区和淮阳产业集聚区为依托，持续打造百亿元电缆电气产业集群。

　　培育发展新兴产业和高成长性产业，扶持发展中小微企业，进一步优化工业结构，提升发展的质量和效益。有针对性地 培育和引进一批装备制造、电子信息等产业项目，支持以富士康、凯旺电子等企业为主体，建设电子信息产业园。实施高新技术企业发展倍增计划，培育一批有较强实力和核心竞争力的创新型企业。落实好促进中小企业发展的政策措施，扶持6家中型企业升级为大型企业，促进66家小型企业发展为规模以上企业，带动创办660家以上微型企业，培育一批新的工业经济增长点。

　　（四）着力推进服务业发展优结构

　　把现代服务业作为新的支柱产业，制定发展规划，完善扶持政策，促进发展提速、比重提高、水平提升。

　　围绕建设具有较强辐射带动能力的豫东南区域商贸物流中心，加强四区协作联动，重点推进中心城区服务业发展。川汇区依托华耀商贸物流城和七一路、文昌大道商业街区建设，重点在商贸物流和特色商业发展上，做强做优，打造亮点；开发区依托新火车站、综合客运总站，重点在客货交通运输服务及相关产业发展上，完善功能，扩大规模；东新区依托商务中心区、职教园区建设，重点在仓储配送、批发零售、金融结算、教育培训等产业发展上，制定规划，积极招商；港口物流产业集聚区依托公、铁、水运输优势，重点在港口经济、农产品和矿产资源物流等产业发展上，开拓市场，提高效益。

　　依托各县市商务中心区、特色商业区规划建设，着力构建各具特色的现代服务业体系，增强服务业在县域经济中的支撑能力。规范房地产市场秩序，提升建筑品质，满足群众住房需求。抓住我市被国家商务部确定为“农产品流通和农村市场体系建设试点市”的发展机遇，推进以黄淮农产品物流市场、万果园农产品深加工园、扶沟万里蔬菜交易物流园为骨干的农产品流通体系建设，逐步把我市建成全国重要的农产品物流基地和交易中心。

　　加强信息服务基础设施建设，促进“三网融合”，加快现代信息技术在服务业中的推广应用。继续开展“无线城市”、“智慧城市”、“数字企业”、“智慧旅游”建设，扩大信息服务业规模，推动服务业扩量提质。用现代信息技术装备和改造工业、提升农业、服务城镇化建设，构建覆盖城乡、惠及全民的信息服务体系，促进四化同步融合发展。

　　（五）着力推进新型城镇化强引领

　　坚持以人为本、优化布局、生态文明、传承文化的原则，突出产业为基、就业为本、生计为先，用新理念规划城市、建设城市、治理城市，努力建设生产空间集约高效、生活空间宜居适度、生态空间天蓝水绿的新型现代城市体系。

　　重点推进中心城区“十大建设”，加快城乡一体化示范区（周口新区）、商务中心区和特色商业区、港口物流产业集聚区建设，掀起中心城区会战热潮，进一步增强中心城区的综合承载能力和辐射带动能力。在全面抓好事关中心城区发展的“四个一批”项目的基础上，重点抓好一批城市综合体、商业综合体项目，推进城区水系改造、沙颍河城区段治理等一批城市基础设施项目。启动引黄调蓄工程建设，建成投用东外环路颍东路段、周口综合客运总站，实施文昌大道提升工程，拆除老火车站，打通并改造提升八一路。集中改建一批小街小巷、公厕、垃圾中转站、污水管网等民生项目，新建10个“两小市场”，加快公交场站建设，着力解决城市占道经营和交通堵塞问题。改造城区供水、供电管网，着力解决城区供水压力不足、部分电网设施老化等问题，提高城区供水、供电保障水平。加大城中村（棚户区）改造力度，将现存城中村全部纳入改造范围，包装项目、分步实施。坚持征迁与安置并重，加快安置房建设，确保拆得掉、搬得进、干得成、见效果。以创建省级文明城市为抓手，完善管理机制，落实管理责任，实施精细化管理，努力改变“脏、乱、差、堵”局面。在东新区、港口物流产业集聚区开展土地整理和一级开发试点，统一规划、统一开发，避免形成新的城中村。保持“两违”治理高压态势，推进“两违”专项治理常态化，确保“两违”整治效果。

　　完成组团城市发展规划编制，引导项城、淮阳、西华、商水与中心城区融合发展。推动中心城区与组团县城在基础设施和公共服务方面均等化配置，探索组团城市之间资源流动、信息共享的途径，提高组团城市的综合集聚和承载能力。支持引导太康、郸城、沈丘、扶沟四个支撑县城加快发展，按照近中期30万人口规模的要求，不断提高承接产业转移、人口集聚和带动农村发展的能力。扎实推进35个重点小城镇建设，进一步完善基础设施，培育产业支撑，提升发展质量，着力构建比较完备的、具有周口地域特色的城镇化体系。

　　加大统筹城乡发展力度，稳步推进新农村建设，不断提高城乡一体化发展水平。坚持产城互动、分类指导的原则，把城中村、城郊村和产业集聚区内的村庄有序纳入城市统一规划、建设和管理，加快城镇化改造。落实农民工入户城镇制度，切实解决农民进城就业、住房、社会保障、子女入学等问题，有序推进农业转移人口市民化，逐步实现城镇基本公共服务常住人口全覆盖。

　　（六）着力推进开放招商增后劲

　　始终坚持对外开放主战略，大力实施招商引资，积极承接产业转移，以开放促改革、促转型、促发展。

　　坚持把招商引资作为主动力，进一步创新招商方式，充分发挥各级工商联、商会和企业在招商引资中的作用，推动从政府主导招商向企业主体招商转变。围绕省、市重点项目,支柱产业和主导产业培育，依托现有企业，紧盯世界500强、国内100强、行业10强和央企，大招商、招大商，促进链式和集群发展。抢抓产业向中西部转移的发展机遇，突出重点区域、重点领域、重点行业，采取划区招商、定点招商等方式，提高招商工作的针对性、有效性和专业化水平。坚持招商和选商并重，采取分产业、差异化的招商策略，更加注重引进竞争力强、成长性强、关联度强的项目，更加注重引进投资强度高、税收贡献率高、人员就业率高、行业知名度高的项目，切实提高招商引资的质量和水平。

　　积极拓宽开放领域，推进对外合作，大力引进先进的技术、人才、理念、机制、管理，力争在农业、服务业、基础设施和社会事业等领域引进一批战略合作项目。支持有条件的企业走出去创业发展，鼓励企业强强联合、合作发展，开拓新市场，提升竞争力。抓好重点企业、重点产品出口，优化出口结构，发展服务贸易，拓展外需市场，保持出口稳定增长。

　　优化投资发展环境，积极营造“重商、亲商、安商、富商”的社会氛围，树立对外开放的良好形象。完善招商引资信息公共服务平台，促进信息资源共享，为外来投资提供高效便捷的服务。深化“转创”活动，严厉查处“吃拿卡要报”和“不作为、乱作为、慢作为”等违规、违纪、违法行为，严厉打击强买强卖、强装强卸、强包工程等损害经济发展环境的不法行为，切实保护好投资者的合法权益。

　　（七）着力推进可持续发展保生态

　　牢固树立保护环境就是保护生产力，改善生态就是改善民生的理念，加强环境保护，建设生态文明，造福子孙后代。

　　重点实施“三大工程”，改善环境质量，努力解决危害群众健康的突出问题。实施“蓝天工程”，建立重污染天气监测预警体系，积极推进城市燃煤锅炉改造、机动车尾气治理、农作物秸秆禁烧、城市施工和道路扬尘防治，落实好大气污染防治各项措施，不断改善空气质量。实施“碧水工程”，加大对沙颍河、运粮河、新蔡河、涡河等流域水环境综合治理力度，依法取缔饮用水源保护区内的违法建设项目，加快污水处理厂等重点减排工程建设，进一步改善水环境质量。实施“乡村清洁工程”，加强农村面源污染治理和土壤污染防治，重点治理规模化畜禽养殖污染，推进农村环境综合整治，完成生态村、生态乡镇创建任务，着力建设美丽乡村。

　　以创建全国绿化模范城市为抓手，加强林业生态建设，不断改善生态环境。围绕中心城区绿化，重点抓好城市森林、廊道绿化、城镇社区绿化、农田林网、森林公园建设五大工程。实施绿色富民提升工程，大力发展花卉苗木、特色经济林、林下种植养殖、林产工业四大林业产业，积极探索生态经济化、经济生态化的发展路子。

　　健全环境保护长效机制，加快转变发展方式。深化主要污染物排放总量控制预算管理，完善污染物排放许可制度，实行最严格的源头保护制度、损害赔偿制度、责任追究制度。严格环保准入和污染物排放标准，继续执行联防联控制度和生态补偿机制。大力发展循环经济，积极发展生态循环农业，提高循环利用水平。倡导绿色生活方式和消费理念，引导公众积极参与，更加自觉地推进绿色发展、循环发展、低碳发展，努力建设美丽周口。

　　（八）着力推进改革创新添活力

　　认真落实各项改革部署，结合我市实际，积极推进有权限、看得准的改革，着力在重点领域、关键环节取得实质性进展。

　　扎实推进财税改革，完善全面规范、公开透明的预算制度。完成国有资产清查登记工作，将所有公共资产的经营收入纳入预算体系，堵塞财政漏洞。加强政府债务管理，有效防范和化解财政风险。认真落实“营改增”扩围等改革政策，加强对税收优惠政策的规范管理，强化依法治税、综合治税。

　　创新金融机构支持地方经济发展的激励机制，继续开展银企对接等金融服务活动，引导金融机构在项目审批、业务权限、信贷投放等方面向我市倾斜。争取部分县农信社组建为农商行，继续推进村镇银行组建工作。创新城市建设融资机制，做好区域集优债、城投债后续发行工作。

　　大力实施科技创新，强化创新驱动。突出企业创新主体地位，落实对企业研发机构的奖励政策，提高企业科技研发积极性，新建一批研发创新平台。坚持质量立市，加快质监检验检测中心和产业集聚区质检服务平台建设，提供技术支撑，提升发展质量。实施一批重大科技专项，促进产学研结合、科技与金融结合、科技与经济结合，完善科技成果转化机制，让科技进步真正成为经济社会发展的驱动力量。

　　创新要素保障机制，满足经济社会发展需求。推动土地管理制度改革，完善土地要素流动机制，着力破解土地供需矛盾难题。建立政府投资公共设施周边土地收储制度，提高政府的调控能力。实施“人地挂钩”试点工作，做好土地计划调控，用好新增建设用地指标、增减挂钩指标，全力保障重点项目建设需要。加大高层次和紧缺人才引进培养力度，在事业单位试行政府购买公共服务的用人办法，不断完善人才保障机制。

　　加快事业单位分类改革，深化国有企业脱钩改制，实施工商注册制度改革，推进文化体制、医药卫生体制等其他重点领域的改革，激发市场主体活力，为经济社会发展注入新的动力。

　　（九）着力推进文化建设促繁荣

　　发挥文化资源优势，创新文化建设载体，激发文化创造活力，不断促进文化旅游融合发展。

　　深入实施公民道德建设工程，积极培育和践行以富强民主文明和谐、自由平等公正法治、爱国敬业诚信友善为基本内容的社会主义核心价值观。开展“做文明人、办文明事”主题实践系列活动，倡导树立文明新风。办好“市民文化大讲堂”等精品文化节目，推动群众性精神文明活动常态化，提高全民素质，凝聚社会正能量。

　　继续实施文化惠民工程，办好“周末公益剧场”等群众文化活动，完善公共文化服务体系，繁荣发展文化事业。实施文化精品工程，创作一批群众喜闻乐见的文艺精品。办好第五届中原古韵中国（淮阳）非物质文化遗产展演活动，做好重要文化遗产和太康道情等非物质文化遗产保护工作。促进哲学社会科学、广播影视、新闻出版、档案事业发展，繁荣文学艺术创作。广泛开展全民健身运动，积极创建全民健身示范市，努力申办2018年河南省第13届运动会。

　　大力发展文化旅游产业，以周家口明清历史文化传承创新为核心，规划建设关帝庙文化休闲产业园区和沙颍河文化休闲景观带，支持关帝庙景区创建国家4A级景区；以太昊陵、龙湖、淮阳古城为核心，高层次规划建设淮阳文化旅游产业集聚区和伏羲文化研究基地，支持羲皇故都、龙湖湿地创建国家5A级景区。挖掘整合文化旅游资源，支持项城袁氏故居、商水叶氏庄园、沈丘槐园、西华女娲城、扶沟吉鸿昌纪念馆等景点的改造提升，做精做优做美一批小景点，不断满足人民群众日益增长的文化娱乐和休闲旅游需求。

　　（十）着力推进民生改善筑和谐

　　继续实施“十项重点民生工程”，抓好服务民生攻坚，加强和创新社会治理，推进和谐社会建设，努力让人民群众过上更好生活。

　　扎实做好就业工作，继续实施全民技能振兴工程和以创业带动就业工程，构建更加完善的公共就业服务体系。年内完成职业技能培训30万人次，新增农村劳动力转移就业10万人。深化收入分配制度改革，拓宽农民增收渠道，持续提高城乡居民收入水平。

　　坚持保基本、兜底线、促公平、可持续，扩大社会保障覆盖范围，健全城乡社会保障体系。完善社会救助制度，提高企业退休职工养老金发放标准和城乡居民低保标准，做好城乡居民养老保险并轨工作。加快实施社会保障“一卡通”。注重解决留守儿童、留守妇女和留守老人生活实际问题。扩大住房公积金覆盖面，加快保障性安居工程建设，继续实施农村危房改造。大力发展社会福利和慈善、老龄、残疾人、红十字等事业，全面提升社会保障水平。

　　继续把教育放在优先发展的战略位置。推进义务教育区域均衡发展和学前教育三年行动计划二期工程，建成投用中心城区“一校两园”，再新建和改扩建1所幼儿园、1所小学、2所中学。各县市都要按照教育改革发展的中长期规划，共同化解城镇“入园难”、“入学难”、“大班额”等突出问题。加强中小学生的国学经典和汉字书法教育，提升素质教育水平。办好高中教育，继续实施周口一高提升工程，力争用5年时间建成河南名校。实施职业教育攻坚工程，积极推进周口职业技术学院“本千万”工程，扎实做好周口科技职业学院“升本”和周口幼师、周口联营职业中专“升专”工作。大力支持周口师范学院转型发展，建设应用型大学，培养汇聚应用型技术人才。高度重视教师队伍建设，及时招录补充教师，加强培训和师德教育，提高业务素质。落实好促进民办教育发展的各项政策，吸引社会资本投资教育事业。

　　加强基层医疗卫生服务体系建设，扎实推进基本公共卫生服务均等化，巩固完善国家基本药物制度和基层运行新机制，稳定乡村医生队伍，持续提高新农合保障水平。扩大中心城区优质卫生资源总量，建成投用市儿童医院，启动建设市中心医院东新区分院。支持和鼓励社会资本举办医疗机构，重点抓好东新区人民医院、周口协和骨科医院等8所民营医院项目建设。加强县级医院能力建设，完善社区卫生服务体系，提高医疗服务水平，着力解决人民群众“看病难”、“看病贵”问题。抓好重大传染病防控救治工作。积极发展中医药事业。稳定低生育水平，完善利益导向政策，提高出生人口素质，促进人口长期均衡发展。

　　继续开展平安周口建设，加强和创新社会治理。注重城市社区建设，加强基层社会管理，依法、有序做好第八届村委会换届选举工作。加强社会诚信体系建设和网络社会管理。开展法制宣传教育，增强全民学法遵法守法用法意识。严格落实安全生产责任，扎实开展道路客货运输、烟花爆竹和消防等重点行业、重点领域的隐患排查整治，坚决遏制较大以上事故发生。建立健全食品药品安全保障体系和监管体系，严格标准、严密监管、严厉惩处、严肃问责，确保人民群众吃得放心、用得安心。加强民族宗教工作，开展民族团结进步创建活动，促进民族经济发展和民族关系和谐。深化信访和社会管理综合治理，做好社会矛盾排查化解工作，强化应急管理体系建设，维护社会大局和谐稳定。

　　继续开展双拥共建，积极创建全国双拥模范城，支持驻周解放军、武警部队、预备役部队、民兵和人民防空建设，促进军民融合式发展。支持工会、共青团、妇联、工商联等人民团体在经济社会发展中发挥桥梁纽带作用。搞好第三次经济普查。继续做好统计、审计、物价、海关、气象、史志、地震、外事、侨务、对口援疆等工作。

　　四、敢于负责、勇于担当，努力建设人民满意的政府

　　新的形势、新的目标、新的任务、新的期盼，对政府工作提出了新的更高要求。我们将始终坚持讲政治、敢担当、勇作为、重实干，切实加强自身建设，着力打造人民满意的政府。在今后的工作中，努力做到：

　　敢于负责，勇于担当。只有牢记责任，才能尽心尽力；只有敢于负责，才养浩然之气。我们将大力倡导和树立敢于负责、勇于担当的精神，直面经济社会发展和改革进程中的困难与问题，切实做到在困难面前，敢闯敢试，决不退缩；在矛盾面前，敢抓敢管，绝不回避；在责任面前，敢作敢为，绝不推诿，切实解决政府系统个别存在的不敢担当、不愿作为的问题，真正把责任扛在肩上，把目标抓在手中，尽心尽力，尽职尽责，干出实实在在的业绩，让自己无愧，让人民满意。

　　少说多做，务实重干。政府工作不在说多少，而在干多少。我们将培育和彰显善始善终、善作善成的实干精神，大力弘扬踏石留印、抓铁有痕的务实作风，察实情、出实招、办实事、求实效，多做基础性、长远性工作，把大事抓实、实事做好，绝不说大话、讲空话、搞形式主义，更不搞脱离实际、劳民伤财的形象工程和政绩工程。切实做到研究工作在一线，分析问题接地气，安排部署有抓手，推进落实见成效，以不达目的绝不罢休的韧劲，努力使政府每一项工作落到实处、见到实效。

　　转变职能，提高效率。打造服务型政府是新形势下政府自身建设的根本任务。我们将认真履行公共服务、市场监管、社会管理、环境保护等职责，切实转变职能，营造良好发展环境，提供优质公共服务，维护社会公平正义。继续开展“两转两提”，清理审批事项，优化简化办事程序，按照环节最少、流程最简、方便企业和群众的原则，将所有政府审批事项和办事流程，在政府网站和行政服务大厅公开。继续实施项目联审联批制度，切实提高办事效率。完成政府机构改革任务，优化机构设置和职能配置，确保财政供养人员只减不增。

　　令行禁止，严明政纪。纪律是执行路线的保证，没有严明的纪律，就难以保证工作任务的落实。我们将严肃行政纪律，严格照章办事。对于中央、省委、省政府和市委的决策部署，必须不折不扣地贯彻执行，言必信、行必果，坚决克服“梗阻”现象，切实解决政策“不落地”问题。凡是市委、市政府研究决定的事情，强化督查落实，绝不允许任何部门、任何单位、任何个人以任何理由推诿扯皮、敷衍塞责、阳奉阴违、顶着不办，真正做到有令则行、有禁则止、政令畅通。

　　依法行政，接受监督。只有坚持依法行政，把权力规范在法律和制度的笼子里，自觉接受监督，才能保证行政权力更加有效地实施。我们将进一步规范行政行为，严格按法定权限和程序履职，深入推进法治政府建设。坚决执行市人大及其常委会的决议、决定，主动接受人大法律监督、工作监督和政协民主监督，进一步做好人大代表建议和政协提案办理工作。认真听取民主党派、人民团体和各界人士的意见建议，继续开展行风评议活动，拓宽群众监督政府的渠道，自觉接受舆论监督、公众监督和社会监督。坚持做到政务公开，重点加强行政审批、工程招投标、土地出让、政府采购等重点领域的监管和审计，决不允许利用身份和权力插手土地的招拍挂和工程的招投标，真正让人民监督权力，让权力在阳光下运行。

　　勤俭节约，廉洁从政。 历览前贤国与家，成由勤俭败由奢。我们将切实负起政府系统廉政建设的主体责任，认真贯彻执行中央“八项规定”，遵守党风廉政建设各项要求，落实《党政机关厉行节约反对浪费条例》，严格控制“三公”经费等一般性支出，把有限的财力优先用在保障和改善民生上，真正做到政府过紧日子，让人民群众过上好日子。按照市委的统一部署，在全市政府系统扎实开展党的群众路线教育实践活动，按照“照镜子、正衣冠、洗洗澡、治治病”的总要求，以严的标准、严的措施、严的纪律，坚决纠正“四风”，树立良好政风。深入推进惩治和预防腐败体系建设，始终做到秉公用权、依法用权、廉洁用权。真正形成有权必有责、用权受监督、失职要问责、违法要追究的良性机制，促进政治清明、政府清廉、干部清正。

　　各位代表！

　　天下之事，行之贵力；为政之要，重在实干。面对历史赋予我们的光荣使命和时代重任，面对全市人民的热切期望和殷殷重托，唯有牢记使命、砥砺奋进，才能实现富民强市、跨越发展；唯有勤勉尽责、毫不懈怠，才能无愧于心，不负千万人民的信任和期盼。时不我待，只争朝夕！让我们紧密团结在以习近平同志为总书记的党中央周围，在省委、省政府和市委的坚强领导下，和衷共济、万众一心，解放思想、开拓创新，努力谱写周口崛起、富民强市、全面建成小康社会的崭新篇章，创造周口更加辉煌的美好明天！
